# Pregnant and Postpartum Women's Experiences and Perspectives on the Acceptability and Feasibility of Copackaged Medicine for Antenatal Care and PMTCT in Lesotho

**DOI:** 10.1155/2015/435868

**Published:** 2015-11-16

**Authors:** Michelle M. Gill, Heather J. Hoffman, Appolinaire Tiam, Florence M. Mohai, Majoalane Mokone, Anthony Isavwa, Sesomo Mohale, Matela Makhohlisa, Victor Ankrah, Chewe Luo, Laura Guay

**Affiliations:** ^1^Elizabeth Glaser Pediatric AIDS Foundation, 1140 Connecticut Avenue NW, Suite 200, Washington, DC 20036, USA; ^2^Department of Epidemiology and Biostatistics, Milken Institute School of Public Health, The George Washington University, 950 New Hampshire Avenue, NW 7th Floor, Washington, DC 20052, USA; ^3^Elizabeth Glaser Pediatric AIDS Foundation, Sechaba House, 1st Floor, 4 Bowker Road, P.O. Box 0166, Maseru West 105, Lesotho; ^4^United Nations Children's Fund, Private Bag A171, Maseru 100, Lesotho; ^5^United Nations Children's Fund, 3 UN Plaza, New York, NY 10017, USA

## Abstract

*Objective.* To improve PMTCT and antenatal care-related service delivery, a pack with centrally prepackaged medicine was rolled out to all pregnant women in Lesotho in 2011. This study assessed acceptability and feasibility of this copackaging mechanism for drug delivery among pregnant and postpartum women.* Methods.* Acceptability and feasibility were assessed in a mixed method, cross-sectional study through structured interviews (SI) and semistructured interviews (SSI) conducted in 2012 and 2013.* Results.* 290 HIV-negative women and 437 HIV-positive women (*n* = 727) participated. Nearly all SI participants found prepackaged medicines acceptable, though modifications such as size reduction of the pack were suggested. Positive experiences included that the pack helped women take pills as instructed and contents promoted healthy pregnancies. Negative experiences included inadvertent pregnancy disclosure and discomfort carrying the pack in communities. Implementation was also feasible; 85.2% of SI participants reported adequate counseling time, though 37.8% felt pack use caused clinic delays. SSI participants reported improvement in service quality following pack introduction, due to more comprehensive counseling.* Conclusions.* A prepackaged drug delivery mechanism for ANC/PMTCT medicines was acceptable and feasible. Findings support continued use of this approach in Lesotho with improved design modifications to reflect the current PMTCT program of lifelong treatment for all HIV-positive pregnant women.

## 1. Introduction

In 2013, 240,000 children were newly infected with HIV, a 58% decline in new pediatric infections since 2002 [1]. There is demonstrated ability to reduce rates of new pediatric HIV infections to less than 5% in Africa, but access to programs for HIV prevention of mother-to-child transmission (PMTCT), uptake, and adherence to drugs are required [2–6].

Literature is replete with documentation of barriers that limit the efficient scale-up and performance of national PMTCT programs. Weak commodity procurement and distribution mechanisms, limited laboratory infrastructure, poor integration of services, limited human resources, and lack of community or patient-centeredness are barriers that limit the health sector's ability to provide the needed services [7]. Challenges with drug stock-outs and monthly dispensing of antenatal Zidovudine (AZT) caused women to make several visits to antenatal care (ANC), potentially deterring them from returning, and thus decreasing PMTCT drug adherence and program effectiveness [8]. A variety of social, behavioral, and structural barriers operate beyond the influence of the health facility, limiting HIV/AIDS services demand from target individuals and communities [9]. Barriers include late ANC presentation, preventing the early initiation of antiretroviral (ARV) drugs for either treatment or prophylaxis, transport costs for women in rural areas, poor retention in services, perceived poor quality of services, lack of support from husbands, lack of privacy, and negative or judgmental attitudes of health workers towards HIV-infected women [8, 10, 11].

In Lesotho, 22.9% are living with HIV and HIV prevalence among pregnant women attending ANC is higher than the national average at 25.9% [1, 12]. Ninety-five percent of pregnant women attend at least one ANC visit. While healthcare engagement is improving, one-quarter of women do not attend the World Health Organization- (WHO-) recommended minimum of four ANC visits nor do they deliver in a facility [13]. As Lesotho is mountainous and the majority of the population lives in rural areas, long distances to clinics, weather conditions, and rough terrain negatively impact the accessibility of critical ANC and PMTCT services. To address some of the operational challenges and decrease MTCT risk for HIV-positive women with limited interactions with the healthcare system, the mother/baby copackage was developed to deliver ARV medications to HIV-positive pregnant women aligned with the WHO guidelines at the time (Option A in 2010) [14].

The mother/baby copackage was adapted from the Minimum PMTCT Package (MPP), implemented in 2007 by the Ministry of Health (MOH) in Lesotho. The MPP contained facility prepackaged drugs in plain envelopes distributed at the first ANC visit for HIV-positive pregnant women and their infants. While both the MPP and copackage were based on a similar drug prepackaging concept, the MPP differed in significant ways including the packaging, contents, and context in which it was provided. The MPP was found to be feasible and acceptable to providers and clients, though challenges such as stock-outs and provision of adequate instruction on the use of multiple medications were noted [15].

**Figure 1:**
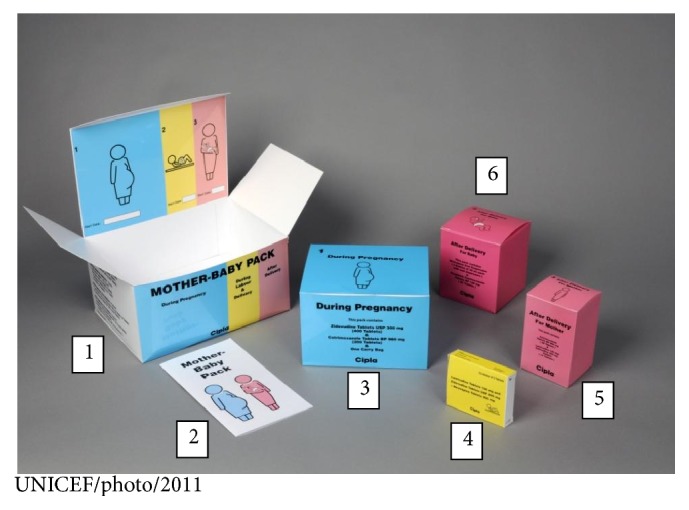
The pack and its inner boxes and instruction sheet. Counterclockwise from the left. (1) Outer pack identical for all women (size: 255 mm × 182 mm × 130 mm). (2) Instruction sheet written in Sesotho. (3) Blue inner box containing medicine to be taken during pregnancy: (i) ferrous sulphate, folic acid, and Vitamin B-complex for all women; (ii) Zidovudine (AZT) for HIV-positive women on ARV prophylaxis. (4) Yellow inner box containing medicine to be taken during delivery: (i) AZT/Lamivudine (3TC) in fixed dose combination and Nevirapine tablets for HIV-positive women on ARV prophylaxis. (5) Pink inner box containing medicine for the mother to be taken postpartum: (i) AZT/3TC 7-day “tail” in fixed dose combination for HIV-positive women on ARV prophylaxis; (ii) vitamin A for all women. (6) Pink inner box containing medicine for the infant through six weeks of age: (i) Nevirapine syrup and syringe for administration for HIV-positive women on prophylaxis and treatment.

The copackage is a color-coded rectangular box, measuring 255 mm × 182 mm × 130 mm (Figure 1). Each pack contained smaller packages inside representing the antenatal, intrapartum, and postpartum periods of pregnancy. The outer bag was also rectangle-shaped and made of dark blue cloth with no words or other markings. To minimize potential stigmatization associated with the pack, three package types were implemented: Pack 1 for HIV-negative women, Pack 2 for HIV-positive women eligible for PMTCT prophylaxis, and Pack 3 for HIV-positive women on antiretroviral treatment (ART). (All packs contained iron, folic acid, Vitamin A, and Vitamin B-complex. In addition, Pack 2 contained AZT tablets from 14 weeks of gestation to delivery and AZT/3TC (fixed dose combination) and Nevirapine tablets for delivery and 7 days postpartum. Providers administering the pack removed excess AZT from the pack based on the gestational age at which the woman received the pack. Pack 2 and Pack 3 also contained Nevirapine syrup with a syringe for administration to the infant until 6 weeks of age.) The packs were assembled and filled centrally and distributed to the health facilities through the existing system. The copackages were administered at the first ANC visit (≥14 weeks of gestation) and contained drugs through six weeks postpartum, including Nevirapine (NVP) for HIV-exposed infants. Pack 3 contained only one month of ART so women on treatment for their own health still had to return monthly to obtain refills. All packs contained ferrous sulphate, folic acid, and Vitamin A and Vitamin B-complex, which were not provided consistently and universally to pregnant women prior to the pack's implementation, and an instruction sheet. All women were advised to bring the pack to each visit in order to provide additional counseling and review of contents and to assess adherence to both the PMTCT drugs and other pack contents. The copackage differed from standard PMTCT care primarily in the drug delivery mechanism: dispensing a supply of maternal and infant drugs at the first ANC visit to last through six weeks postpartum, particularly to target HIV-positive women on prophylaxis in case they did not return for a subsequent ANC visit or deliver in a health facility. However, women were counseled on the importance of returning to the facility for HIV and pregnancy-related care. Women and their infants continued clinic follow-up after six weeks postpartum with collection of additional infant NVP for women on prophylaxis and additional ARV for women on ART. The pack was first piloted in three districts in January 2011 and then rolled out nationally to the remaining seven districts by August 2011.

In April 2013, the country shifted to universal, lifelong ART for all HIV-positive pregnant and breastfeeding women (known as “Option B+”), aligned with the 2013 WHO PMTCT guidelines [16, 17]. The national program continued with the use of Pack 1 and Pack 3. Therefore, it was important to assess how women felt about this novel drug delivery system (acceptability), how the copackage-related services were delivered to women (feasibility), and how future use could be aligned with the simplification of drug supply and distribution systems and the ARV regimens and messaging to women afforded by the Option B+ approach.

## 2. Materials and Methods

This was a mixed method, cross-sectional study. Structured exit interviews (SI) and in-depth semistructured interviews (SSI) with pregnant and early postpartum women were conducted from December 2012 to May 2013.

### 2.1. Study Population and Recruitment

The study was conducted in six purposively selected districts out of the ten districts in Lesotho. Three districts were those selected by the MOH to first implement the mother/baby copackage. Three comparable districts with later initiation were selected to match the three geographical settings of Lesotho (Lowlands, Foothills, and Highlands) represented in the initial implementing districts. Within each district, health facilities (HF) were randomly selected as study sites using the probability proportional-to-size (PPS) method to ensure all HF had the same probability of being selected. SI were conducted in all 31 study sites while SSI were conducted in two study sites per district (highest volume hospital and health center) for a total of 12 sites.

HIV-positive and HIV-negative women who were attending a subsequent ANC visit and 6-week and 14-week postnatal care (PNC) visits at study sites were eligible for participation in interviews. Health care workers (HCW) introduced the study and assisted in linking potential participants with study staff. Study staff attempted to conduct SSI with HIV-positive women who did not return to ANC or early PNC to capture their experiences with the copackage and any influences it may have had on their health-seeking behavior. We used the routine PMTCT program follow-up system to identify and trace women who had missed visits. When these women were found, the community or health worker introduced them to the study and invited them to speak with study staff at a convenient time and location.

### 2.2. Structured Interview Methods

A sample of 196 women in each of the three groups (HIV-negative, HIV-positive on prophylaxis, and HIV-positive on ART) was targeted. We estimated 85% of women would find the intervention both acceptable and feasible, based on the MPP evaluation [15]. Assuming an expected proportion of 0.85, the large sample normal approximation was used to calculate a two-sided 95% confidence interval around the observed proportion with a margin of error of 0.05 to determine a needed sample size for SI of 196. The target sample per HF was determined using PPS based on routinely collected antenatal attendance program data.

Women were selected through a random process by which they were consecutively referred and screened on days when study staff were present, until either the sample size at each HF was reached or the data collection period ended. HF targets were further divided into six subgroups of roughly equal numbers based on all possible combinations of visit type (ANC, PNC) and type of copackage received (Pack 1, Pack 2, and Pack 3). Once a subgroup target was reached, women in that particular group were no longer eligible. Trained maternal and child health (MCH) study nurses explained the study, obtained and documented verbal informed consent, and conducted SI using data collection instruments specifically designed according to the type of visit and type of copackage received. All interviews were conducted in Sesotho. SI involved closed-ended questions, including a series of seven copackage acceptability (e.g., size, convenience, and design) and eight copackage feasibility (e.g., counseling, understanding, and clinic flow) statements. Women were asked to indicate whether they agreed or disagreed or had no opinion for each statement. They were also asked to describe what they liked/disliked about the copackage and their positive/negative experiences related to its use. Interviewers selected precoded responses that best fit the women's answers and documented other responses that could not be classified.

### 2.3. Semistructured Interview Methods

SSI guides covered similar topics, but their open-ended nature allowed interviewers to probe more deeply. These interviews were intended to capture richer and more complex information than the SI. As such, a smaller approximate sample size range was estimated in order to reach saturation of theme: 9–15 HIV-positive women (with approximately equal targets for women on prophylaxis and ART) and 4–6 HIV-negative women per HF for a total of 108–180 and 48–72, respectively, with subgroup targets by visit type (ANC, 6-week PNC, 14-week PNC). SSI study staff were different from those conducting SI and were trained in qualitative research methods. Consecutive recruitment of women on days when study staff were present was similar to the SI process. If SI and SSI recruitment were taking place at the same HF on the same day, women were first approached to participate in the SSI; if they declined, they were asked whether they were willing to participate in the shorter SI. SSI were audio recorded and were simultaneously transcribed and translated into English. Transcripts were reviewed by the study coordinators and/or investigators once translated.

### 2.4. Data Analysis

**Figure 2:**
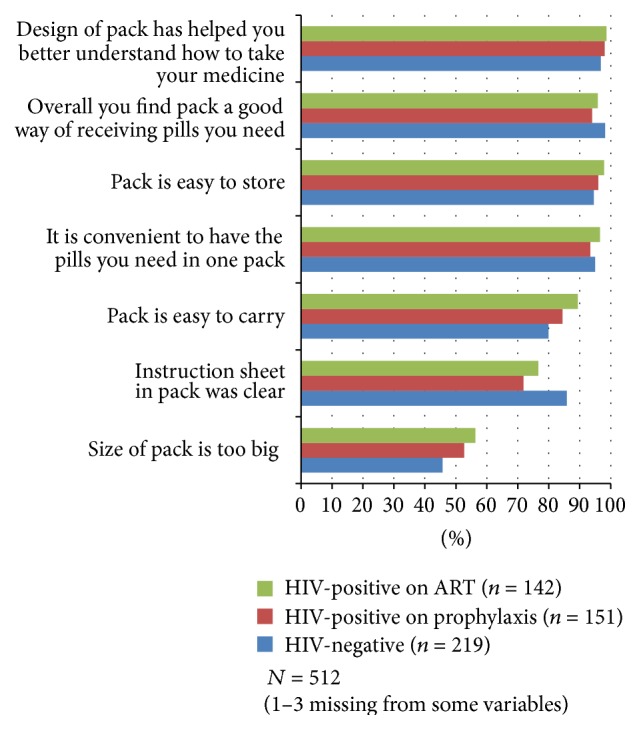
Percentage of structured interview participants that agree with the copackage acceptability statements by HIV status group. (Possible responses to statements included agree, disagree, and no opinion. “No opinion” responses ranged from 0.4 to 2.0% of the three groups combined for each statement, except the instruction sheet statement, in which 13.7%, 24.8%, and 22.0% of HIV-negative, HIV-positive on prophylaxis, and HIV-positive on ART women responded with “no opinion,” because they did not have or did not use the sheet.)

**Figure 3:**
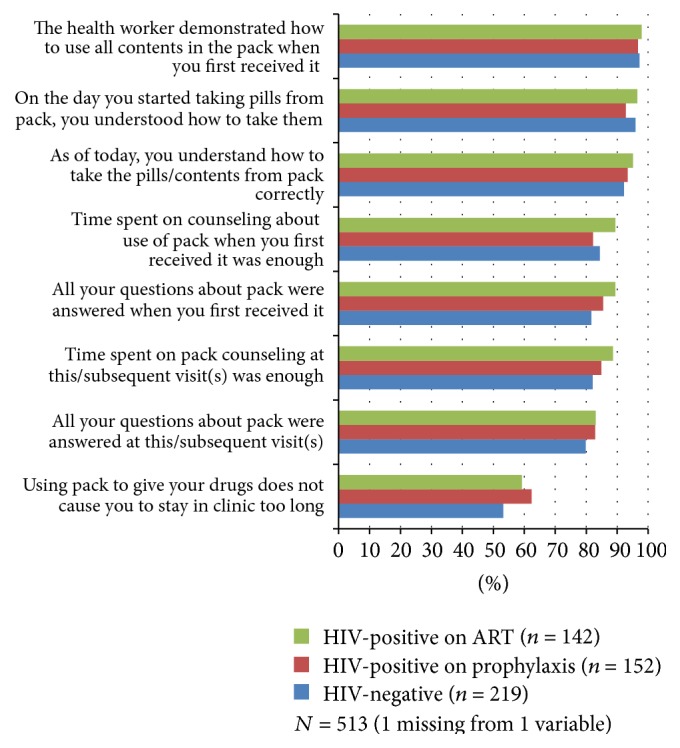
Percentage of structured interview participants that agree with the copackage feasibility statements by HIV status group. (Possible responses to statements included agree, disagree, and no opinion. For most statements, “no opinion” responses ranged from 0.6 to 7.0% of the three groups combined for each statement. The exceptions were “all questions about the pack were answered” in which 12.8%, 10.5%, and 8.5% of HIV-negative, HIV-positive on prophylaxis, and HIV-positive on ART women, respectively, responded with “no opinion,” at first visit and 16.1%, 13.2%, and 12.7%, respectively, responded with “no opinion,” at this visit (if interviewed in ANC) or subsequent visits (if interviewed in PNC) because they did not have questions.)

Data from close-ended questions in SI and SSI were entered into a Microsoft Access database (2007) using a double data entry and verification system. Descriptive statistics were calculated and reported for key characteristics of the study participants and agreement with acceptability and feasibility statements and reported experiences. Means and standard deviations were reported for all continuous variables (Table 1) and frequencies and percentages were reported for all categorical variables (Tables 1 and 2 and Figures 2 and 3). Differences in (1) the level of agreement with acceptability and feasibility statements and (2) the experience of either positive or negative consequences as a result of pack receipt among HIV-negative women, HIV-positive women on prophylaxis, and HIV-positive women on ART were examined with generalized estimating equations (GEE) using the binomial distribution with the logit link for dichotomous outcomes or the multinomial distribution with the cumulative logit link for ordinal outcomes. Select significant findings are highlighted in the text. Compound symmetry and independent working correlation structures were considered to account for the clustering of women in multiple facilities. Score tests were used to test the proportional odds assumption, and the Tukey-Kramer method for *P* value adjustment was used to account for multiple comparisons. All statistical tests were two-sided and the level of statistical significance was set at 0.05. All data were analyzed in Washington, DC, USA, using SAS/STAT software version 9.3 (Cary, North Carolina).

SSI transcripts were imported into MAXqda (V10). A codebook was created based on the research objectives and variables of interest. Data were coded by a team in the US and Lesotho and reviewed for consistency by one investigator. Textual data were carefully reviewed to identify recurrent patterns and themes and draw conclusions connected to the study questions. Data were summarized through descriptive, text-based summaries and data display matrices. Both deductive codes based on research questions and inductive codes derived from the data were utilized.

### 2.5. Ethical Approval

This study was approved by the George Washington University Institutional Review Board (IRB), the Baylor College of Medicine Lesotho IRB, and the Lesotho MOH ethical review committee. All participants underwent a verbal informed consent process in Sesotho using an IRB-approved verbal consent text with documentation of consent by study staff.

## 3. Results

### 3.1. Study Population

523 women participated in SI and 204 women participated in SSI (Table 1). Overall, 348 women were interviewed in ANC and 379 in PNC. 290 were HIV-negative and 437 were HIV-positive (*n* = 224 on prophylaxis, *n* = 202 on ART, *n* = 11 with unknown regimen) with a mean age of 26.6 years. Women with unknown regimen did not receive a pack; they were asked close-ended demographic and pregnancy-related questions only since they could not address questions on copackage acceptability and feasibility. The majority of women (87.0%) received the pack at their first ANC visit; 16 women (2.2%) did not receive the pack. Among pregnant women who participated in SI and SSI in ANC, 91.3%, 94.4%, and 87.5% of HIV-negative, HIV-positive women on prophylaxis, and HIV-positive on ART, respectively, brought the pack with them to the ANC visit when the interview was conducted.

Attempts were made to locate 79 women who were identified as clinic defaulters. 45 women were found; of whom 16 were still in care and were misclassified as defaulters. Of the other 29, eight were deemed ineligible by study staff, two declined to be interviewed, and 14 did not arrive for their interview appointments (information was missing for one woman). Of the four who arrived for their interview appointments, study staff failed to conduct the interview for one woman, one woman was determined ineligible after interviewing, and two interviews were conducted. Among the other 34 women, 23 could not be found, nine had relocated, and two were deceased.

### 3.2. SI Acceptability

Nearly all (96.3%) SI participants found the copackage to be acceptable (Figure 2). There were high levels of agreement across all three groups (HIV-negative, HIV-positive on prophylaxis, and HIV-positive on ART) with the acceptability statements. Half (50.7%) of participants felt the size of the pack was too big. Significantly more women on ART (89.4%) felt the pack was easy to carry than HIV-negative women (79.9%, OR = 0.47, 95% CI: 0.30, 0.74, *P* < 0.0003). (The intraclass correlation coefficients (ICCs) for acceptability and feasibility statement comparisons across groups were all negligible (<0.01).) The instruction sheet, meant to be included in each pack to provide supplemental guidance on use, was not used or missing from the pack in 13.7%, 24.8%, and 22.0% of reports by HIV-negative, HIV-positive on prophylaxis, and HIV-positive on ART women, respectively. A missing or unused instruction sheet was reported by significantly more women on prophylaxis compared to HIV-negative women (OR = 2.43, 95% CI: 1.33, 4.45, *P* < 0.0017). Supplemental Table 1, in Supplementary Material available online at http://dx.doi.org/10.1155/2015/435868, provides the GEE results (*P* values, ORs and 95% CIs) for each acceptability statement by HIV status group.

### 3.3. SSI Acceptability

Women participating in SSI also found the copackage acceptable but agreed the packs were too big and often referred to it as luggage. They recommended reducing the pack's size so that it could fit in a handbag. In order for packs to appear identical, all packs contain the same inner boxes, though some were empty for HIV-negative and HIV-positive women on ART, but this feature made the packs seem unnecessarily large to some women:

*The pack should be smaller… again another reason it is because some of my boxes were empty so I was saying that I do not see a point of being given such a big empty box. (HIV-positive woman on ART in PNC)*

Women made recommendations to improve pack design and its transport, including adding a strap so that it can be carried as a handbag. Labeling the diagrams on the pack flap in Sesotho (not English); ensuring the instruction sheet is included in pack and placed prominently inside; and writing the dosage on pill bags and bottles and ordering them by month so women would understand to finish one before opening the next one were other recommendations to improve the pack.

### 3.4. SI Positive and Negative Experiences

While 17.7% of women reported no positive experiences using the pack, the majority (77.8%) reported no negative experiences (Table 2). The most-frequently mentioned positive experience by 35.0% of women was that the pack helped them to take their pills as instructed. In total, only 7.3% of women indicated that the pack allowed them to attend clinic less often. The most common negative experience reported by 16.3% of women was that the pack identified women as pregnant before they were ready to disclose. In contrast, 8.8% of women considered being identified as pregnant by the pack a positive experience. HIV-negative women were more likely to report identification of pregnancy as a positive experience (13.7%) than HIV-positive women on prophylaxis (2.7%, OR = 5.30, 95% CI: 1.91, 14.72, *P* < 0.0004). Three instances of ill-treatment by partners who discovered women's HIV-positive status because of the pack were reported. In GEE models, there were no statistically significant differences in those who reported any positive pack-related experience (87.4%, 82.7%, and 79.4%, *P* = 0.17) or any negative experience (22.0%, 25.5%, and 21.6%, *P* = 0.65) among the HIV-negative, HIV-positive on prophylaxis, and HIV-positive on ART groups, respectively.

### 3.5. SSI Positive and Negative Experiences

Receipt of all pills at once and the health-promoting qualities of the pack and contents were the aspects women most liked about the pack. The most commonly cited positive experiences were that the pack or drug contents promoted healthy pregnancies and deliveries; partners or family members accepted and supported women using pack; and women felt happy or excited when walking to/from the clinic with the pack. One woman describes going home with the pack after she first received it:

*It was good. It's because I think this pack and pills will help my baby to be born healthy. I was happy. (HIV-positive woman on prophylaxis in ANC) *

The fact that all packs have identical outer packaging helped some women regardless of status to feel comfortable carrying the pack and deflect any attention or questions about their pack, simply by responding that all pregnant women received them. While a few women reported rumors in the community that the pack was associated with HIV, particularly during early months of implementation, most people came to realize that the copackage was provided to all women seeking antenatal services.

*I like the pack because all people are given the same bag and no one will see if you are given HIV drugs. It enables one's status to be known to that person alone. (HIV-positive women on prophylaxis in PNC)*

Similar to SI, pregnancy identification was one of the most frequently mentioned dislikes, as well as heeding the advice of nurses to carry the pack with them everywhere. There were reports from both HIV-negative and HIV-positive women that they hid the pack in another bag when in public.

*I do not like carrying the pack, I put it in my bag because I do not want people to see that I am pregnant and know that whenever I am carrying the pack, I am going to the clinic even though my pregnancy is not visible. (HIV-negative woman in PNC)*

Overall, the frequency of negative experiences reported because of the pack was low. These included feelings of discomfort or shame when carrying the pack in the community; inadvertent disclosure of HIV status when community members incorrectly perceived that all women with the pack were HIV-positive; and being mocked by community members when carrying the pack. There were also few problems reported with women's partners or mothers-in-law as a result of receiving the pack. While not related to the pack itself, some women did not like the supplements in the packs as they felt taking them would result in large infants that would make delivery more difficult. Negative experiences were reported by one of the two women who missed visits. The woman felt stigmatized by a neighbor who assumed she was HIV-positive after being seen with the pack. She also reported initial confusion due to all of the information provided during initial copackage counseling. However, neither woman reported receipt of the copackage as the reason for missing visits.

### 3.6. SI Feasibility

There were high levels of agreement with feasibility statements among all three groups and any differences were statistically nonsignificant (Figure 3). 95.1% of women understood how to take pills from the pack from the first day it was used and 93.4% understood on the day of the interview. Time spent on counseling at visit when pack was first received as well as at subsequent visits was considered adequate by 85.2% and 84.8% of women, respectively. Approximately 38% felt use of the pack caused clinic delays. Supplemental Table 2 provides the GEE results (*P* values, ORs and 95% CIs) for each feasibility statement by HIV status group.

### 3.7. SSI Feasibility

Most women said that it was easy for them to take medication from the pack. Reported drug-taking errors were rare (e.g., continuing AZT prophylaxis after delivery, taking the wrong supplement dosage). SSI participants described longer counseling sessions when the pack was first dispensed, but that counseling was helpful and the length was appropriate to the topics being discussed. They also generally felt their questions were adequately addressed during counseling. Women were counseled that the purpose of the pack was to promote mother and child health and prevent MTCT, though there was some difficulty in understanding the purpose of the empty boxes and carrying it to each visit, particularly for HIV-negative women. For instance, some HIV-negative and HIV-positive on ART women had not been counseled to understand that they had an empty delivery box and why (this medication was only included in the HIV prophylaxis pack). During counseling, some women reported that nurses referred to the information sheet without explaining it and were told to read it on their own or it was not mentioned at all. Those that used the sheet found it helpful. Few women reported little or no counseling at follow-up visits.

Most SSI participants who had attended ANC for previous pregnancies agreed waiting times increased following the introduction of the copackage, though quality of services had improved due to more education and counseling and adherence assessments. When asked if services have changed with pack introduction, one woman responded:

*They have changed a lot because this time we have been given such help…I realized that we get services in a good way. You are examined, whether you like it or not you have to be examined for the sake of your baby…back then when they see you complaining they left you without examining you- they did the other small things and left you- but now we are cared for. (HIV-positive woman on ART in PNC)*

Women perceived the counseling and time spent at the clinic to be longer than previously experienced. However, most women appreciated the time at clinic because they felt they were receiving more comprehensive services: they received pills that they had not received previously and they were counseled on the entire course of pregnancy and beyond and on the importance of the drugs contained in the pack. Their pack contents were also physically checked instead of simply asking women if they had taken their drugs.

The majority of women in SSI preferred to receive all of their pregnancy and early postpartum medications in the pack at one time, largely because they felt it would be difficult to return to clinic by the exact day that their pills finished (e.g., due to travel, lack of transport, or transport money). However, one respondent described the reason for her preference was that it helped understand the importance of the medicine:

*I want it to be given once…If we are given it packet after packet – we will not get the benefit of the pack like the picture so that we are able to learn about them, and the instruction sheet and the boxes in here. We cannot be able to know about it, I think when I take that one packet and go home with it I will not know anything about the benefits of this pack. (HIV+ woman on ART in ANC)*

## 4. Discussion

Overall, women felt that the copackage was an acceptable and feasible way of receiving HIV-related and safe motherhood medicines during pregnancy and early postpartum in Lesotho. Women expressed high levels of agreement with statements related to overall pack acceptability as well as the ease to which the pack was stored, carried, and used to take medicine. Women also agreed with statements regarding sufficient counseling and understanding of the pack and its contents. There were few negative experiences associated with pack and most women expressed a preference to receive prepackaged medicines. Despite overall acceptance, there were suggestions noted for improvement to pack design, largely related to the pack's size, to draw less attention to women carrying the pack and to improve ease of its transport.

Other evaluations of prepackaged medicines had similarly favorable findings. There were high levels of acceptability and utilization of contents among mothers in Pakistan provided with a prepackaged intervention, including oral rehydration salts and Zinc tablets distributed for home use and aimed at diarrhea treatment for their young children [18]. A “mama kit” implemented in Uganda containing preventative malaria treatment was also accepted by the pregnant women to whom it was administered [19].

The pack inadvertently disclosed women's pregnancies earlier than some would have liked, irrespective of HIV status, though women did not indicate that this prevented them from attending clinic. In contrast, Andrew et al. found that some women, particularly those stigmatized for being pregnant, such as adolescents or single women, were discouraged from attending ANC, since they felt that doing so would disclose their pregnancies to the community [20].

The improvement in quality of services many women attributed to the pack may have been because the pack was accompanied by a change in counseling that focused more individual attention on women and the importance of “soft” skills in counseling in addition to the messaging. Ugandan “mama kits” were accompanied by midwife training that included customer care and how to provide a friendly environment. Mbonye et al. found that among the significant factors influencing adherence to treatment was promise of the pack and kind midwives, highlighting the importance of friendly services that help women feel cared for and valued [19]. Authors concluded that a patient-centered approach over authoritarian or paternalistic approaches to counsel pregnant women to stop smoking in South Africa was preferred as it helped to facilitate better relationships between midwives and patients [21]. Positive feedback from nurses, among other factors, aided in effective communication with patients during family planning consultations in Indonesia [22].

Few women reported attending clinic less often due to the copackaged medicines dispensed at the first ANC visit. Therefore the pack does not appear to be a barrier to the WHO recommendation of attending at least four ANC visits, based on this limited finding. However, differences in the delivery and uptake of services using the pack compared with provision of the Option A regimen without the pack were investigated retrospectively using routinely collected data and will be presented elsewhere. It is not possible to discern from these results the extent to which the pack addressed the issue of providing sustained medication to the group of women who only attended one ANC visit. In addition, women who did not return to ANC after pack receipt may not have found the pack as acceptable and feasible compared to women regularly attending care. While efforts were made to gather experiences from women who did not return for subsequent visits, there was limited representation from this group. Given that the copackage was rolled out nationwide in Lesotho by the time of the evaluation, it was not possible to have a comparison group of women who did not receive the pack. However, multigravida women in SSI provided valuable responses in their comparisons of pregnancy-related experiences at the clinic preceding and following pack introduction.

Another study limitation is that there may be a social desirability bias skewing women's responses in a positive direction. Women who responded with “no opinion” to any of the acceptability and feasibility statements (e.g., for women who did not have questions to be answered during counseling) were included in the denominator, but not the numerator, for the percentage calculation of women who agreed with these statements. Therefore, the percentages are conservative estimates for most positive statements and would be higher if these responses were removed from the denominator.

While it was not part of this evaluation, an assessment of the costs would be an important undertaking for national program planners to make a decision about long-term implementation of the pack. Providing the copackage to all women is more costly than targeting HIV-positive women. However, these findings indicate that having identical packs dispensed to all women did contribute to women's acceptability of the pack and that the benefits of the pack extend beyond those with an HIV-positive diagnosis. In addition, the initial copackaging was designed and produced externally, which is considerably more costly than having the revised packs designed, produced, and filled locally. Reducing the size of the pack and eliminating the inner boxes which are no longer necessary under the Option B+ program would help to minimize pack costs. The pack size was based on the group of women requiring the greatest number of contents, pregnant women on ARV prophylaxis, which is no longer relevant under the current PMTCT approach. The simplification of treatment regimens under Option B+ is aligned with a primary purpose of the pack, to simplify drug distribution and delivery systems. Moreover, Option B+ implementation could be enhanced by the prepackaging and counseling aspects of the pack.

## 5. Conclusions

This study found that an innovative prepackaged drug delivery mechanism is a feasible and acceptable alternative to traditional ANC and PMTCT drug dispensing. Women generally liked the pack, which may be attributed in large part to more comprehensive counseling and perceived improvement in quality of care that accompanied its use. Suggested improvements to the size and design of packs incorporated with modifications needed to reflect the current Option B+ regimen should further increase both the acceptability and feasibility of this mechanism. Results from this evaluation have significant implications for the PMTCT program in Lesotho, as well as globally, as other countries consider implementation of similar copackaging mechanisms for integrated drug distribution to maximize the health of women and children.

## Supplementary Material

Supplemental Table 1 presents the GEE results for the comparisons among women in each HIV group by their response to statements on pack acceptability. Supplemental Table 2 presents the GEE results for the comparisons among women in each HIV group by their response to statements on pack feasibility. The results include the omnibus test for significance for each HIV group (p-values only) and the partial tests comparing each group's responses to each other group's responses on whether or not they agreed with statements about the pack (p-values, ORs and 95% CIs).

## Figures and Tables

**Table 1:** Characteristics of study participants.

	Structured interview (SI) participants	Semistructured interview (SSI) participants	Total
Total *N* (%)	523	204	727

Continuous variable	*N*	*N*	*N*
	Mean (SD)	Mean (SD)	Mean (SD)

Age	522	201	723
	26.6 (6.3)	26.5 (6.0)	26.6 (6.2)
Gravida	520	203	723
	2.1 (1.3)	2.3 (1.4)	2.2 (1.3)

Categorical variable	*N* (%)	*N* (%)	*N* (%)

Districts			
Qacha's Nek	26 (5.0)	36 (17.7)	62 (8.5)
Mafeteng	125 (23.9)	43 (21.0)	168 (23.1)
Butha Buthe	51 (9.8)	31 (15.2)	82 (11.3)
Berea	173 (33.1)	35 (17.2)	208 (28.6)
Mohale's Hoek	86 (16.4)	35 (17.2)	121 (16.6)
Mokhotlong	62 (11.9)	24 (11.8)	86 (11.8)
Status/regimen			
HIV-negative	223 (43.1)	67 (33.7)	290 (40.5)
HIV-positive/prophylaxis	152 (29.4)	72 (36.2)	224 (31.3)
HIV-positive/ART	142 (27.5)	60 (30.2)	202 (28.2)
HIV-positive/unknown regimen^*∗*^	6	5	11
Visit			
ANC	262 (50.1)	86 (42.2)	348 (47.9)
PNC	261 (49.9)	118 (57.8)	379 (52.1)
ANC visit pack received			
1st	456 (87.7)	174 (85.3)	630 (87.0)
2nd	48 (9.2)	20 (9.8)	68 (9.4)
3rd	7 (1.3)	2 (1.0)	9 (1.2)
≥4th	1 (0.2)	0 (0)	1 (0.1)
Did not receive pack	8 (1.5)	8 (3.9)	16 (2.2)
Unknown^*∗*^	3	0	3
Number of ANC visits (PNC only)			
1	9 (3.8)	5 (4.6)	14 (4.0)
2	32 (13.4)	13 (11.9)	45 (12.9)
3	61 (25.5)	32 (29.4)	93 (26.7)
4	69 (28.9)	28 (25.7)	97 (27.9)
>4	68 (28.5)	31 (28.4)	99 (28.4)
Unknown^*∗*^	19	9	28
Did not attend ANC^*∗*^	3	0	3

^*∗*^Excluded from the percentage calculation.

**Table 2:** Women's positive and negative experiences with the pack reported in structured interviews.

*N* (%)	HIV-negative (*n* = 219)	HIV-positive/prophylaxis (*n* = 148)	HIV-positive/ART (*n* = 142)	Total (*n* = 509)
What positive experiences have you had with the pack?				
Pack motivated to disclose to spouse	41 (18.7)	32 (21.6)	22 (15.5)	95 (18.7)
Pack motivated to disclose to family	14 (6.4)	22 (14.9)	9 (6.3)	45 (8.8)
Received support from partner after disclosure	47 (21.5)	40 (27.0)	34 (23.9)	121 (23.8)
Received support from family member after disclosure	8 (3.7)	26 (17.6)	15 (10.6)	49 (9.6)
Led to unplanned disclosure with acceptance from partner	5 (2.3)	6 (4.1)	3 (2.1)	14 (2.8)
Community treated me better with pack	13 (5.9)	9 (6.1)	13 (9.2)	35 (6.9)
Motivated me attend clinic to have healthy baby	31 (14.2)	30 (20.3)	34 (23.9)	95 (18.7)
Allowed me to attend clinic less often	21 (9.6)	10 (6.8)	6 (4.2)	37 (7.3)
Helped take all the pills as instructed	83 (37.9)	54 (36.5)	41 (28.9)	178 (35.0)
Identified me as pregnant	30 (13.7)	4 (2.7)	11 (7.8)	45 (8.8)
Drug contents improved mother/child health	17 (7.8)	6 (4.1)	5 (3.5)	28 (5.5)
No positive experiences	30 (13.7)	28 (18.9)	32 (22.5)	90 (17.7)

*N* (%)	HIV-negative (*n* = 218)	HIV-positive/prophylaxis (*n* = 149)	HIV-positive/ART (*n* = 142)	Total (*n* = 509)

What negative experiences have you had with the pack?				
Partner found out status and was verbally abused	0	1 (0.7)	0	1 (0.2)
Partner found out status and was treated badly	0	1 (0.7)	0	1 (0.2)
Partner found out status and was abandoned	0	1 (0.7)	0	1 (0.2)
Family member found out status and was treated badly	0	2 (1.3)	3 (2.1)	5 (1.0)
Confused about pack and drugs	0	1 (0.7)	1 (0.7)	2 (0.4)
Difficult because I had to hide pack	1 (0.5)	3 (2.0)	2 (1.4)	6 (1.2)
Having drugs in pack made me forget to take drugs	1 (0.5)	1 (0.7)	0	2 (0.4)
People treated me badly when they saw pack	2 (0.9)	3 (2.0)	3 (2.1)	8 (1.6)
Others suspected/discovered HIV+ status	1 (0.5)	1 (0.7)	4 (2.8)	6 (1.2)
Identified me as pregnant	40 (18.3)	25 (16.8)	18 (12.7)	83 (16.3)
No negative experiences	171 (78.4)	112 (75.2)	113 (79.6)	396 (77.8)
