# Supplementary material for: Pregnant and Postpartum Women's Experiences and Perspectives on the Acceptability and Feasibility of Copackaged Medicine for Antenatal Care and PMTCT in Lesotho
Source: AIDS Res Treat. 2015 Nov 16;2015:435868. doi: 10.1155/2015/435868 (PMC4663282; doi:10.1155/2015/435868)
Supplement: Supplementary file 1 — Supplemental Table 1 presents the GEE results for the comparisons among women in each HIV group by their response to statements on pack acceptability. Supplemental Table 2 presents the GEE results for the comparisons among women in each HIV group by their response to statements on pack feasibility. The results include the omnibus test for significance for each HIV group (p-values only) and the partial tests comparing each group's responses to each other group's responses on whether or not they agreed with statements about the pack (p-values, ORs and 95% CIs). [file 435868.f1.docx]

**Supplemental Table 1. Model outcomes as agree vs. no opinion vs. disagree with the co-package acceptability statements by HIV status group**

| **Statement** | **HIV-negative**  **N (%)** | **HIV-positive on prophylaxis**  **N (%)** | **HIV-positive on ART**  **N (%)** | **Total**  **N (%)** | **p-value***  **OVERALL**  **NEG v. ART**  **NEG v. PROPHY**  **ART v. PROPHY** | **OR (95% CI)***  **NEG v. ART**  **NEG v. PROPHY**  **ART v. PROPHY** |  |
| --- | --- | --- | --- | --- | --- | --- | --- |
| The pack is easy to carry  Agree  No opinion  Disagree | 175 (79.9)  4 (1.8)  40 (18.3) | 129 (85.4)  3 (2.0)  19 (12.6) | 127 (89.4)  1 (0.7)  14 (9.9) | 431(84.2)  8 (1.6)  73(14.3) | 0.0004  **0.0003**  0.14  0.30 | **0.47 (0.30, 0.74)**  0.67 (0.41, 1.10)  1.43 (0.82, 2.50) |  |
| The pack is easy to store  Agree  No opinion  Disagree | 207 (94.5)  2 (0.9)  10 (4.6) | 145 (96.0)  0 (0)  6 (4.0) | 139 (97.9)  0 (0)  3 (2.1) | 491 (95.9)  2 (0.4)  19 (3.7) | 0.09  0.07  0.78  0.48 | 0.37 (0.13, 1.07)  0.72 (0.23, 2.26)  1.92 (0.51, 7.20) |  |
| The size of the pack is too big  Agree  No opinion  Disagree | 100 (45.7)  2 (0.9)  117 (53.4) | 79 (52.7)  3 (2.0)  68 (45.3) | 80 (56.3)  1 (0.7)  61 (43.0) | 259 (50.7)  6 (1.2)  246 (48.1) | 0.07  0.12  0.08  0.75 | 0.65 (0.39, 1.08)  0.74 (0.53, 1.03)  1.13 (0.76, 1.69) |  |
| It is convenient to have the pills you need in one pack  Agree  No opinion  Disagree | 208 (95.0)  3 (1.4)  8 (3.6) | 141 (93.4)  0 (0)  10 (6.6) | 137 (96.5)  1 (0.7)  4 (2.8) | 486 (94.9)  4 (0.8)  22 (4.3) | 0.26  0.77  0.77  0.23 | 0.69 (0.20, 2.38)  1.36 (0.47, 3.94)  1.97 (0.74, 5.21) |  |
| The design of the pack has helped you better understand how to take your medicine  Agree  No opinion  Disagree | 212 (96.8)  4 (1.8)  3 (1.4) | 148 (98.0)  0 (0)  3 (2.0) | 140 (98.6)  1 (0.7)  1 (0.7) | 500 (97.7)  5 (1.0)  7 (1.4) | 0.46  0.58  0.74  0.94 | 0.43 (0.06, 3.08)  0.62 (0.14, 2.83)  1.43 (0.12, 17.17) |  |
| The instruction sheet in the pack was clear  Agree  No Opinion  Disagree | 188 (85.8)  30 (13.7)  1 (0.5) | 107 (71.8)  37 (24.8)  5 (3.4) | 108 (76.6)  31 (22.0)  2 (1.4) | 403 (79.2)  98 (19.3)  8 (1.6) | 0.0024  0.05  **0.0017**  0.51 | 1.86 (0.99, 3.46)  **2.43 (1.33, 4.45)**  1.31 (0.74, 2.32) |  |
| Overall, you found the MBP a good way of receiving the pills you needed  Agree  No opinion  Disagree | 214 (98.2)  1 (0.5)  3 (1.4) | 142 (94.0)  3 (2.0)  6 (4.0) | 136 (95.8)  6 (4.2)  0 | 492 (96.3)  10 (2.0)  9 (1.8) | 0.15  0.42  0.13  0.65 | 2.30 (0.49, 10.79)  3.41 (0.77, 15.18)  1.48 (0.52, 4.23) |  |

*Obtained from GEE models using the multinomial distribution with the cumulative logit link and an independent working correlation structure. ORs (95% CIs) and p-values in order from top to bottom correspond to the omnibus test for significance of the HIV group variable (p-values only), partial test comparing negative v. HIV-positive on ART, partial test comparing negative v. HIV positive on prophylaxis, and partial test comparing HIV-positive on ART v. HIV positive on prophylaxis. The Tukey-Kramer method for p-value adjustment was used to account for multiple comparisons.

**Supplemental Table 2. Model outcomes as agree vs. no opinion vs. disagree with the co-package feasibility statements by HIV status group**

| **Statement** | **HIV-negative** | **HIV-positive**  **on prophylaxis** | **HIV-positive**  **on ART** | **Total** | **p-value***  **OVERALL**  **NEG v. ART**  **NEG v. PROPHY**  **ART v. PROPHY** | **OR (95% CI)***  **NEG v. ART**  **NEG v. PROPHY**  **ART v. PROPHY** |
| --- | --- | --- | --- | --- | --- | --- |
| Time spent on counseling about use of the pack when you first received it was enough  Agree  No opinion  Disagree | 184 (84.4)  6 (2.8)  28 (12.8) | 125 (82.2)  1 (0.7)  26 (17.1) | 127 (89.4)  3 (2.1)  12 (8.5) | 436 (85.2)  10 (2.0)  66 (12.9) | 0.11  0.37  0.72  0.09 | 0.64 (0.29, 1.39)  1.20 (0.69, 2.10)  1.88 (0.93, 3.79) |
| All your questions about the pack were answered when you first received it  Agree  No opinion  Disagree | 178 (81.7)  28 (12.8)  12 (5.5) | 130 (85.5)  16 (10.5)  6 (4.0) | 127 (89.4)  12 (8.5)  3 (2.1) | 435 (85.0)  56 (10.9)  21 (4.1) | 0.12  0.11  0.41  0.46 | 0.52 (0.24, 1.12)  0.75 (0.44, 1.27)  1.44 (0.70, 2.97) |
| The health worker demonstrated how to use all of the contents in the pack when you first received it  Agree  No opinion  Disagree | 212 (97.2)  3 (1.4)  3 (1.4) | 147 (96.7)  1 (0.7)  4 (2.6) | 139 (97.9)  0 (0)  3 (2.1) | 498 (97.3)  4 (0.8)  10 (1.9) | 0.77  0.92  0.93  0.76 | 0.77 (0.17, 3.55)  1.21 (0.35, 4.24)  1.57 (0.36, 6.94) |
| On the day you started taking pills from the pack, you understood how to take the pills/contents from the pack  Agree  No opinion  Disagree | 209 (95.9)  2 (0.9)  7 (3.2) | 141 (92.8)  1 (0.7)  10 (6.6) | 137 (96.5)  0 (0)  5 (3.5) | 487 (95.1)  3 (0.6)  22 (4.3) | 0.22  0.97  0.37  0.38 | 0.86 (0.18, 4.07)  1.82 (0.64, 5.22)  2.13 (0.56, 8.14) |
| Time spent on MBP counseling *at this visit/subsequent visits* was enough  Agree  No opinion  Disagree | 179 (82.1)  22 (10.1)  17 (7.8) | 129 (84.9)  9 (5.9)  14 (9.2) | 126 (88.7)  5 (3.5)  11 (7.8) | 434 (84.8)  36 (7.0)  42 (8.2) | 0.17  0.15  0.85  0.65 | 0.61 (0.33, 1.13)  0.85 (0.42, 1.72)  1.39 (0.58, 3.34) |
| All your questions about the pack were answered *at this visit/subsequent visits*  Agree  No opinion  Disagree | 174 (79.8)  35 (16.1)  9 (4.1) | 126 (82.9)  20 (13.2)  6 (3.9) | 118 (83.1)  18 (12.7)  6 (4.2) | 418 (81.6)  73 (14.3)  21 (4.1) | 0.66  0.66  0.82  1.00 | 0.81 (0.47, 1.42)  0.82 (0.38, 1.77)  1.01 (0.48, 2.12) |
| As of today, you understand how to take the pills/contents from the pack correctly  Agree  No opinion  Disagree | 201 (92.2)  6 (2.8)  11 (5.1) | 142 (93.4)  2 (1.3)  8 (5.3) | 135 (95.1)  1 (0.7)  6 (4.2) | 478 (93.4)  9 (1.8)  25 (4.9) | 0.54  0.52  0.93  0.77 | 0.62 (0.22, 1.73)  0.84 (0.29, 2.47)  1.36 (0.48, 3.81) |
| Using the pack to give all of your drugs to you does not cause you to stay in the clinic too long  Agree  No opinion  Disagree | 116 (53.2)  12 (5.5)  90 (41.3) | 94 (62.3)  8 (5.3)  49 (32.4) | 84 (59.2)  4 (2.8)  54 (38.0) | 294 (57.5)  24 (4.7)  193 (37.8) | 0.11  0.70  0.12  0.62 | 0.82 (0.46, 1.46)  0.69 (0.44, 1.07)  0.84 (0.54, 1.30) |

*Obtained from GEE models using the multinomial distribution with the cumulative logit link and an independent working correlation structure. ORs (95% CIs) and p-values in order from top to bottom correspond to the omnibus test for significance of the HIV group variable (p-values only), partial test comparing negative v. HIV-positive on ART, partial test comparing negative v. HIV positive on prophylaxis, and partial test comparing HIV-positive on ART v. HIV positive on prophylaxis. The Tukey-Kramer method for p-value adjustment was used to account for multiple comparisons.
